# The One Note System: Implementation and Initial Perceptions of Student Documentation in the Electronic Health Records Under the New Centers for Medicare and Medicaid Services Guidelines

**DOI:** 10.7759/cureus.9702

**Published:** 2020-08-12

**Authors:** Komal Safdar, Evan M Dombrosky, Claire Kimberly, Rebecca Miller, Adam M Garber, Steven Bishop, Marieka Helou

**Affiliations:** 1 Internal Medicine, Virginia Commonwealth University, Richmond, USA; 2 Pediatrics, Virginia Commonwealth University, Richmond, USA

**Keywords:** student documentation, cms guidelines, patients over paperwork, electronic health record, note bloat

## Abstract

Introduction

Medical students have been documenting notes in the electronic health records (EHR) for many years but often wrote separate notes from housestaff and faculty because licensed providers (LPs) could not bill the Centers for Medicare and Medicaid Services (CMS) for Evaluation and Management (E/M) services. However, in 2018, CMS updated its policy to allow LPs to simply verify any component of an E/M service under appropriate supervision, allowing LPs to bill a full medical student note.

Methods

At Virginia Commonwealth University Health Systems (VCUHS), a task force was formed to develop and pilot the One Note System (ONS), a system that incorporates the new CMS guidelines for certain note types. In June 2019, or 10 months after implementation of the ONS, the authors developed and distributed a survey that explored perceptions regarding the ONS among medical students, housestaff (residents and fellows), and faculty.

Results

The results showed that most participants were aware of the ONS and preferred email as the form of training. Overall, the ONS had a positive impact on faculty and housestaff workflow, improved self-reported faculty wellbeing, and increased meaning in student work. Only a minority reported barriers to implementing the ONS.

Conclusions

The One Note System was successfully implemented at VCUHS and positively received. Other outcomes to measure include impact of the ONS on student and trainee education, compliance and billing, quality and quantity of documentation, and faculty and housestaff burnout rates.

## Introduction

Documenting clinical encounters is a critical physician skill. The Association of American Medical Colleges (AAMC) emphasizes its importance by including it as one of the 13 core entrustable professional activities (EPAs), which are skills all medical graduates should be trusted to perform with indirect supervision on day one of intern year [1]. Medical students have been typing notes in electronic health records (EHRs) for several years. However, in 2018 the Centers for Medicare and Medicaid Services (CMS) changed the Evaluation and Management (E/M) documentation policy by adding CR 10412 to the Medicare Claims Processing Manual. This new CMS policy allows licensed providers (LPs) to simply verify any component of an E/M service directly observed and subsequently documented by a student in the medical record [2]. If a resident is the LP, final verification is still required from a teaching physician. The term “verify” is key in that the LP no longer needs to write an addendum to student notes or re-document their work; under appropriate direct observation, they can edit the original student note as their own. Prior to this new guideline, LPs could only bill CMS for student documentation of past medical/social/family/surgical history and review of systems. 

The new guideline is part of the CMS "Patients Over Paperwork Initiative," which is aimed at reducing unnecessary administrative burdens on practitioners [2]. A time and motion study in the ambulatory setting showed that physicians spend two hours in the EHR for every one hour spent with patients during work hours, and that physicians spend an additional one to two hours each night to complete clerical work [3]. Similarly, internal medicine interns spend only 12-17% of their time in direct patient care and more than 40% of their time is spent on a computer, of which documentation is one of the most time-consuming tasks [4]. Indeed, a survey of over 16,000 trainees demonstrated that most residents spent more than four hours a day solely on documentation [5]. This overwhelming time burden can contribute to a phenomenon known as “note bloat,” in which outdated information is copied-forward leading to too much irrelevant information within a single note and difficulty finding pertinent information [6]. A survey of 351 physicians showed that 90% of physicians used the copy and paste function and 71% of those physicians acknowledged an increase in inconsistencies or outdated information in notes [7].

With these considerations, the new CMS guidelines have the potential to change the landscape for student involvement in documenting clinical care as part of the official health record and ultimately to enhance the quality of documentation. Currently, it is unclear how many medical schools have adopted the new CMS guidelines. At Virginia Commonwealth University Health Systems (VCHUS), we implemented a new “One Note System" (ONS), which incorporates the new guidelines. Here we describe our implementation process and the results of a survey assessing faculty, residents/fellows, and student perceptions of the new ONS at our institution. 

## Materials and methods

Program details

After the new CMS guidelines were released, VCU Health System (VCUHS) created a task force of physicians and compliance officers to develop and pilot a new system for documentation incorporating these new guidelines. Training included the development and distribution of a “Job Aid” outlining the steps in the EHR with applicable screenshots as well as individual presentations and discussion at each clinical department meeting by a member of the task force. 

In the new ONS, medical students are allowed to initiate and contribute to billable documentation. If a medical student initiates the note, the history and physical examination must have been performed in the physical presence of an LP (residents/fellows or attending), who must fully review and edit the note as appropriate. If the supervising LP is housestaff (resident/fellow), the attending physician must also fully review and edit the housestaff note. If a note is initiated by housestaff alone, the attending physician must only repeat the critical and/or key portions of the physical exam and confirm the medical decision making. The attending physician could then review/edit and sign the housestaff note without additional addendums. All contributors to these notes are required to place a pre-populated attestation specific to their level of training, which outlines their roles and responsibilities within the note.

These changes for medical students applied to initial and established outpatient encounters, inpatient consults, and inpatient progress notes. During the pilot phase, Admission History and Physicals (H&Ps) and Discharge Summaries were excluded from the ONS. Participants underwent training in the ONS prior to initiation in August of 2018.

Survey development

We developed a survey (created and distributed via RedCap© [8]) to explore perceptions regarding the implementation of the ONS and adoption of new CMS guidelines among faculty, housestaff, and students. The survey asked participants about their clinical role (i.e. faculty, fellow, resident, student), clinical specialty, work setting (i.e. inpatient, outpatient, or both), if they were aware of the ONS, and their perceptions of its impact. The response choices available for perceived impact of the ONS were on a five-point scale ranging from significant negative impact to significant positive impact. The survey ended with an open-ended question asking about additional concerns or unforeseen consequences. To ensure content validity, a small focus group (10) of faculty, housestaff, and medical students piloted the survey. The researchers made edits to the survey based on the feedback from these participants before dissemination.

Setting and participants

We distributed the survey to all teaching faculty, housestaff, and third-year medical students at the VCUHS and Virginia Commonwealth University School of Medicine (VCU SOM) 10 months after initial implementation of the ONS. Responses were anonymous and survey participation was voluntary. There was no compensation for participating and no penalties for not participating. Data were securely stored and presented in aggregate form to the institution. This study was deemed exempt by the Institutional Review Board of VCU.

## Results

A total of 317/1573 (20.2%) of participants started the survey and 224 completed it; only completed responses were included in this analysis. The participants were mostly faculty (n = 118; 52.7%), followed by students (n = 64; 28.5%), residents (n = 32; 14.4%), and fellows (n = 10; 4.5%). The most common departments represented were internal medicine (n = 83; 36.9%), pediatrics (n = 30; 13.4%), and emergency medicine (n = 17; 7.6%); the additional 16 departments had less than 10 participants each. A total of 84.5% (n = 98) of faculty and 77.6% (n = 90) of fellows, residents, and students were aware of the One Note policy. Only these participants were prompted to complete the rest of the survey (n = 165), which included 22 questions on the impact of the policy on their profession or position and overall well-being. A summary of the results can be found in Table 1. 

**Table 1:** Perceptions of faculty, housestaff, and students on the One Note System at VCUHS in 2019. Abbreviations: Virginia Commonwealth University Health System (VCUHS) Participants were asked on a five-point scale (with 1 = significantly negative, and 5 = significantly positive) how much the One Note System impacted each statement listed. Negative is represented by the selection of “1 or 2,” neutral is represented by the selection of “3,” positive is represented by the selection of “4 or 5.”

Question	Position	Negative %(n)	Neutral %(n)	Positive %(n)	I don’t know %(n)
The time for you to complete your daily documentation.					
	Faculty	1.3(1)	9.2(7)	85.5(65)	4.0(3)
	Fellow	0.0(0)	0.0(0)	100.0(6)	0.0(0)
	Resident	8.7(2)	13.0(3)	78.3(18)	0.0(0)
	Student	13.3(8)	36.7(22)	35.0(21)	15.0(9)
Time for you to practice writing notes.					
	Fellow	0.0(0)	66.7(4)	16.7(1)	16.7(1)
	Resident	0.0(0)	60.9(14)	30.4(7)	8.7(2)
	Student	6.7(4)	5.0(3)	88.3(53)	0.0(0)
Time for clinical/bedside teaching.					
	Faculty	0.0(0)	46.7(35)	49.3(37)	4.0(3)
	Fellow	0.0(0)	0.0(0)	83.3(5)	16.7(1)
	Resident	0.0(0)	47.8(11)	47.8(11)	4.4(1)
	Student	5.0(3)	58.3(35)	28.3(17)	8.3(5)
Your satisfaction with the impact on the time of day you complete/submit your documentation					
	Faculty	4.0(3)	24.0(18)	68.0(51)	4.0(3)
Impact on your daily documentation workflow					
	Faculty	2.7(2)	24.0(18)	70.7(53)	2.7(2)
The amount of extraneous information in any one progress note in the EMR					
	Faculty	9.3(7)	34.7(26)	53.3(40)	2.7(2)
	Fellow	16.7(1)	16.7(1)	66.7(4)	0.0(0)
	Resident	13.0(3)	52.2(12)	34.8(8)	0.0(0)
	Student	18.3(11)	20.0(12)	51.7(31)	10.0(6)
The number of redundant progress notes for any given patient on any given date					
	Faculty	1.3(1)	13.3(10)	78.7(59)	6.7(5)
	Fellow	0.0(0)	0.0(0)	100.0(6)	0.0(0)
	Resident	0.0(0)	0.0(0)	100.0(23)	0.0(0)
	Student	15.0(9)	6.7(4)	75.0(45)	3.3(2)
Vulnerability to compliance infractions					
	Faculty	20.0(15)	44.0(33)	22.7(17)	13.3(10)
Concern about medico-legal risk					
	Faculty	21.3(16)	54.7(41)	14.7(11)	9.3(7)
Opportunities for interns to develop documentation skills					
	Faculty	6.7(5)	34.7(26)	48.0(36)	10.7(8)
	Fellow	0.0(0)	16.7(1)	50.0(3)	33.3(2)
	Resident	8.7(2)	39.1(9)	43.5(10)	8.7(2)
Preparedness for residency compared to the traditional student documentation process					
	Student	1.7(1)	11.7(7)	80.0(48)	6.7(4)
The accuracy of the patient notes					
	Faculty	9.3(7)	30.7(23)	58.7(44)	1.3(1)
	Fellow	0.0(0)	33.3(2)	66.7(4)	0.0(0)
	Resident	0.0(0)	47.8(11)	47.8(11)	4.4(1)
The conciseness of the patient notes					
	Faculty	13.3(10)	30.7(23)	54.7(41)	1.3(1)
	Fellow	50.0(3)	0.0(0)	50.0(3)	0.0(0)
	Resident	17.4(4)	52.2(12)	26.1(6)	4.4(1)
Engagement/patient ownership on the part of the medical students					
	Faculty	0.0(0)	12.0(9)	80.0(60)	8.0(6)
	Fellow	0.0(0)	0.0(0)	100.0(6)	0.0(0)
	Resident	0.0(0)	4.4(1)	91.3(21)	4.4(1)
	Student	0.0(0)	5.0(3)	95.0(57)	0.0(0)
Sense of meaning in your daily work ('my work counts')					
	Student	0.0(0)	1.7(1)	98.3(59)	0.0(0)
Sense of meaning in students' daily work ('their work counts')					
	Faculty	0.0(0)	8.0(6)	81.3(61)	10.7(8)
	Fellow	0.0(0)	0.0(0)	100.0(6)	0.0(0)
	Resident	0.0(0)	4.4(1)	82.6(19)	13.0(3)
Impact on my wellness					
	Faculty	1.4(1)	32.4(24)	62.2(46)	4.0(3)
	Fellow	0.0(0)	33.3(2)	66.7(4)	0.0(0)
	Resident	0.0(0)	34.8(8)	60.9(14)	4.4(1)
	Student	3.3(2)	48.3(29)	46.7(28)	1.7(1)
Frequency of faculty reviewing student-generated notes					
	Faculty	4.0(3)	22.7(17)	69.3(52)	4.0(3)
	Fellow	0.0(0)	0.0(0)	16.7(1)	83.3(5)
	Resident	0.0(0)	8.7(2)	60.9(14)	30.4(7)
	Student	1.7(1)	11.7(7)	83.3(50)	3.3(2)
Feedback to medical students on their notes					
	Faculty	2.7(2)	21.3(16)	72.0(54)	4.0(3)
	Fellow	0.0(0)	0.0(0)	100.0(6)	0.0(0)
	Resident	0.0(0)	8.7(2)	87.0(20)	4.4(1)
	Student	1.7(1)	18.3(11)	75.0(45)	5.0(3)

Faculty were further asked about which training they received; the most popular was receiving email instructions about the ONS (n = 54; 24.3%). This was followed by in-person training (n = 32; 14.4%), department-wide training (n = 31; 14.0%), and division-wide training (n = 26; 11.7%). Only 6.7% (n = 11) were not satisfied with the training process. 

Upon asking about barriers about the ONS, the survey prompted participants to select if they were concerned about any of the following: general education concerns, workflow for residents and faculty, problems related to billing, private insurance payer concerns, and medical/legal litigation concerns. Less than 3% of participants selected that they had any of the listed concerns.

While the current One Note policy excluded H&Ps and Discharge Summaries, 92.6% (n = 151) of participants still felt that H&Ps should be included in the new policy and 76.1% (n = 124) believed that discharge summaries should be included. Responses were nearly evenly split on whether these responsibilities should be extended to only M4s, or M3s and M4s. Although not a significant difference, faculty were more likely to state that H&Ps should not be included in the One Note policy when compared to the opinions shared by residents/fellows (6.8% versus 3.4%, respectively). Similarly, faculty were less likely to state that the One Note policy should be expanded to writing discharge summaries when compared to residents/fellows (24.3% and 31.0%, respectively).

## Discussion

Our institution successfully implemented the new CMS student documentation guidelines for many common note types. A core group of physician and administrative champions were key to the success of this project and spent a significant amount of time working with our partners in compliance to ensure the new process was correctly integrated into the health system. Additionally, this initial core group performed most of the hands-on training for faculty, housestaff, and students to ensure the process was followed properly. Our data supports that the vast majority were satisfied with the training; emailed instructions were the most utilized method.

The survey responses report an overwhelmingly positive impact on faculty and housestaff at our institution. A total of 85.5% (n = 65) of faculty and 78.2% (n = 18) of residents noted a positive impact on the time it took to complete daily documentation, a primary goal sought out by the new CMS policy change. A total of 82.2% (n = 46) of faculty and 60.9% (n = 14) of residents also noticed a positive effect on wellness. These findings are consistent with the handful of studies that describe administrative burden as a driver towards physician burnout [9] though our low response rate limits the generalizability of our results. Regardless, burnout negatively impacts many factors including workplace productivity and efficiency, and quality of patient care and patient safety [10]. While we did not intend to study burnout, our data supports that the new CMS guidelines could help move us in the direction of reduced physician burnout and improved patient care. 

The potential benefits of the new CMS guidelines also extend to students. Our data show that 75.0% (n = 45) of students and 72.0% (n = 54) of faculty felt that faculty are providing feedback more often to trainees with the new policy. Prior to these new guidelines, there was little incentive for providers to observe and confirm student exams or review student notes given it often added to the heavy workload of teaching physicians, who already review resident notes. In addition, 46.7% (n = 28) of students reported a positive impact on their wellness, and nearly every student felt a greater sense of meaning in their daily work (n = 59; 98.3%) and increased patient engagement/patient ownership (n = 57; 95.0%). This is consistent with Hammound et al. who observed that the simple act of writing patient notes can lead to an improved sense of ownership of the patient and professional responsibility as part of a team [11].

Lastly, most participants noted a reduction in redundant notes in patient charts. This is intuitive given that there is one note instead of three separate accounts of the patient encounter. The majority of faculty (n = 44, 58.7%) also perceived increased accuracy of patient notes. While we did not assess quality or accuracy of notes, the One Note policy has the potential to decrease the erroneous information being included in the chart and make coding and billing less challenging. This is an area that deserves further investigation in future studies. 

There are several limitations to this preliminary data. First, the residents/fellows and faculty results should only be extrapolated to the internal medicine and pediatrics services as responses from other departments were minimal. This might be due to the fact that these departments utilize the ONS more than others based upon the type of documentation required and emphasized in different departments. In addition, while the survey design allowed for some refinement in the pilot stage, we have no formal validity data for its use outside of this setting. Due to the significant proportion of individuals who started the survey but did not finish it, there are concerns about its length and ease of navigating. Lastly, these results represent only the perceptions of a small cohort of physicians, trainees, and students at one institution.

## Conclusions

VCUHS is now over one year into the new process and ONS implementation and will be expanding to allow students to initiate Admission H&Ps in the coming months. While we will continue to monitor the results of the policy change, our data supports that the new CMS guidelines can have many benefits for LPs, housestaff, and medical students. Additional steps needed to study the change include evaluating outcomes related to: student and trainee education, compliance and billing, quality of documentation in the medical record (i.e. if those notes include more accurate and higher yield data than previously found in routine patient documentation), and quantity of documentation in the medical record in order to determine if there are fewer notes (less duplicative documentation).
